# Revisiting Diabetic Ketoacidosis (DKA) Fluid Management: Should Normal Saline Be Used?

**DOI:** 10.7759/cureus.77739

**Published:** 2025-01-20

**Authors:** Noelle Messina, Zackary Anderson, Lauren Saravis, Glendy Jimenez, Keegan Plowman, Doug Harrington

**Affiliations:** 1 Graduate Medical Education Internal Medicine Residency, Naples Comprehensive Health (NCH) Healthcare System, Naples, USA; 2 Graduate Medical Education Pulmonary and Critical Care Residency, Naples Comprehensive Health (NCH) Healthcare System, Naples, USA; 3 Division of Pulmonary Critical Care Medicine, Naples Comprehensive Health (NCH) Healthcare System, Naples, USA; 4 NCH Internal Medicine Residency Program, Naples Comprehensive Health (NCH) Healthcare System, Naples, USA

**Keywords:** anion-gap metabolic acidosis, diabetic ketoacidosis (dka), isotonic saline, ketotic hyperglycemia, lactated ringers, volume replacement

## Abstract

Background

Diabetic ketoacidosis (DKA) is a common and serious complication of diabetes, often requiring hospitalization and intensive care. Fluid resuscitation is a cornerstone of DKA management, with traditional guidelines recommending isotonic normal saline (NS) for initial volume replacement. Recent studies, however, suggest that large volumes of NS may lead to undesirable outcomes such as hyperchloremic metabolic acidosis. This study investigates the effects of large-volume NS resuscitation on clinical outcomes in DKA management, comparing it to other fluids, such as lactated Ringers (LR).

Objective

To evaluate whether large-volume resuscitation with isotonic normal saline (NS) is associated with prolonged ICU length of stay (LOS), increased time on insulin infusion, and higher rates of non-anion gap metabolic acidosis in patients with DKA.

Materials and methods

This was a single-center, retrospective, observational study conducted at Naples Comprehensive Healthcare System. We reviewed electronic medical records of patients diagnosed with DKA, defined by pH <7.3, bicarbonate <18, and anion gap >12. The primary outcome was ICU LOS, and secondary outcomes included overall length of stay, insulin infusion duration after DKA resolution, and incidence of non-anion gap metabolic acidosis after DKA resolution. Patients were grouped by the amount of NS received during resuscitation: 0L, 1L, 2L, and ≥3L. Statistical analyses included analysis of variance (ANOVA), t-tests, and chi-square tests to compare outcomes between groups.

Results

A total of 109 patients were included in the study. The mean age was 51.34 years, and the cohort consisted of 43.1% females and 56.9% males. There was no significant difference in ICU LOS between patients who received 0L and 1L of NS. However, patients who received 2L (p=0.0249) and ≥3L (p=0.00065) had significantly longer ICU LOS compared to those who received 0L of NS. No significant difference in overall LOS was also observed across all groups (p=0.894). Patients who received ≥3L of NS had a significantly longer duration of insulin infusion compared to those who received 0L (p=0.0101) after DKA anion gap closure while a significant increase in the incidence of non-anion gap acidosis after DKA resolution was observed in patients receiving ≥2L of NS (p=0.0000).

Conclusion

This study suggests that large-volume resuscitation with isotonic NS in DKA patients is associated with increased ICU length of stay, prolonged insulin infusion, and a higher incidence of non-anion gap metabolic acidosis. These findings support the use of balanced crystalloids, such as lactated Ringers, for initial resuscitation in DKA patients, as they may reduce the risk of complications related to hyperchloremia and improve clinical outcomes. Further prospective studies are needed to confirm these findings and guide fluid management protocols in DKA.

## Introduction

Diabetic ketoacidosis (DKA) is a complication of uncontrolled hyperglycemia driven by absolute insulin deficiency in diabetic patients. It has also been reported in euglycemic patients taking SGLT-2 inhibitors [1]. It has an incidence of 56 per 100,000 people annually [2]. Although a serious and life-threatening condition, DKA is a preventable complication of diabetes. Despite being preventable, the Centers for Disease Control and Prevention (CDC) proposes this complication has become more common from 2009 to 2014, with an increase in hospitalization of nearly 6% during this period. In-hospital mortality rates have decreased over the same time frame [2]. Standardized protocols have been developed to ensure uniform treatment of this preventable complication [3]. The gold standard of treatment for DKA focuses on insulin, electrolyte supplementation, and fluid resuscitation. Traditionally, large-volume resuscitation with isotonic saline has been utilized to achieve a euvolemic state [4]. This practice has come under scrutiny due to recent studies demonstrating an increased incidence of hyperchloremic non-anion gap acidosis in patients receiving large volumes of isotonic saline [5]. One retrospective analysis showed that lactated Ringers led to a shorter duration of acidosis with less incidence of non-anion gap acidosis than normal saline [6]. This calls into question the ideal fluid for resuscitation in DKA patients who typically present severely hypovolemic due to osmotic diuresis from hyperglycemia [7]. This study aims to retrospectively evaluate whether large-volume resuscitation with normal saline led to a longer ICU length of stay, overall length of stay, increased time on insulin infusion, and increased incidence of non-anion gap metabolic acidosis after resolution of DKA.

## Materials and methods

This is a single-center, retrospective observational study. Institutional review board (IRB) approval was obtained from the Naples Comprehensive Healthcare System (NCH) IRB. No written consent was required, as this was a retrospective study. Electronic medical record data were collected for clinical purposes, and no additional tests or interviews were necessary. All data were de-identified and stored in Health Insurance Portability and Accountability Act (HIPAA)-secured environments. A list of patients was generated through the electronic medical record (EMR) using a list of ICD 10 codes [8], including E09.10 (drug or chemical-induced diabetes mellitus with ketoacidosis without coma), E09.11 (drug or chemical-induced diabetes mellitus with ketoacidosis with coma), E13.10 (other diabetes mellitus with ketoacidosis without coma), E13.11 (other diabetes mellitus with ketoacidosis with coma), E10.10 (type 1 diabetes mellitus with ketoacidosis without coma), E10.11 (type 1 diabetes mellitus with ketoacidosis with coma), E11.10 (type 2 diabetes mellitus with ketoacidosis without coma), E11.11 (type 2 diabetes mellitus with ketoacidosis with coma), E08.10 (diabetes due to underlying condition with ketoacidosis without coma), and E08.11 (diabetes due to underlying condition with ketoacidosis with coma). This resulted in a list of 280 patients over the course of a two year time period. A manual chart review was then conducted on each patient. Patients were excluded if they were younger than 18 years old, if they were not in diabetic ketoacidosis, or if they were not placed on an insulin infusion.

Patient charts were manually reviewed to collect age at the time of admission, sex, race, initial glucose, initial anion gap, initial bicarbonate, initial chloride, initial creatinine, initial potassium, anion gap on DKA anion gap closure, bicarbonate on DKA anion gap closure, chloride on DKA anion gap closure, creatinine on DKA anion gap closure, non-anion gap metabolic acidosis on DKA gap closure, time in DKA, time on insulin infusion, duration on insulin infusion after DKA gap closure, amount of normal saline given as resuscitation bolus, amount of lactated Ringers given as resuscitation bolus, total amount of intravenous fluids given as resuscitation bolus, ICU length of stay, and overall length of stay. The primary author was responsible for data oversight and maintaining the integrity of the data. Quality control was performed throughout data collection and statistical analysis. The data supporting this study's findings are available upon reasonable request from the corresponding author.

Per our hospital protocol, a patient is in DKA if the pH is decreased, the anion gap is greater than 12, and the bicarbonate is less than 18. DKA is said to be resolved when the patient meets three criteria: pH greater than 7.3, bicarbonate greater than or equal to 18, and an anion gap less than or equal to 12. The amount of fluid given for initial fluid resuscitation in patients with DKA was evaluated. The amount of normal saline, lactated Ringers, and total IVF in liters was recorded. The number of liters of normal saline that were given for resuscitation was recorded, and patients were separated into four groups: normal saline 0 liters (NS 0), normal saline 1 liter (NS 1), normal saline 2 liters (NS 2), and normal saline 3 or greater liters (NS 3-4). There were only four patients who received 4L of normal saline, and they were included in the 3 liter or greater group, as there were not enough patients to analyze as their own group. ICU length of stay was measured in hours. It was calculated using admission time to the hospital and time transferred from the ICU. Insulin infusion time was calculated in hours. The duration of insulin infusion after DKA anion gap closure was calculated once the DKA anion gap was closed. Charts were reviewed to determine if patients had a non-anion gap metabolic acidosis after the DKA anion gap closed. This was defined as a bicarbonate level less than 22 after anion gap levels were 12 or less. The primary endpoint was the ICU length of stay. Secondary endpoints include overall length of stay, duration of insulin infusion after DKA anion gap closure, and non-anion gap metabolic acidosis on DKA anion gap closure.

Patients were divided into their respective groups based on the amount of normal saline given for initial resuscitation. Analysis of variance (ANOVA) was then performed on each group to evaluate its relationship with ICU length of stay. A t-test for two independent means was used to evaluate each individual group in comparison to each other and ICU length of stay. The secondary endpoint of the length of stay was evaluated using one-way ANOVA. The secondary endpoint of duration on insulin infusion after DKA anion gap closure was evaluated using the t-test for 2 independent means. The secondary endpoint of non-anion gap metabolic acidosis was evaluated using chi-square analysis.

## Results

Of the initial 280, 171 patients were excluded, leaving a total of 109 patients included in the study. The exclusion for these patients was because they were not in DKA based on the parameters set.

Baseline characteristics

**Table 1:** Baseline characteristics of patients

SEX	n (%)
Female	47 (43.1%)
Male	62 (56.9%)
RACE	n (%)
Asian	1 (0.9%)
African American	16 (14.7%)
White	76 (69.7%)
Other	16 (14.7%)
AGE, MEAN, +/- SD (RANGE)	51.34 +/- 19.387 (18 - 87)

Primary endpoint

ICU Length of Stay

**Figure 1:**
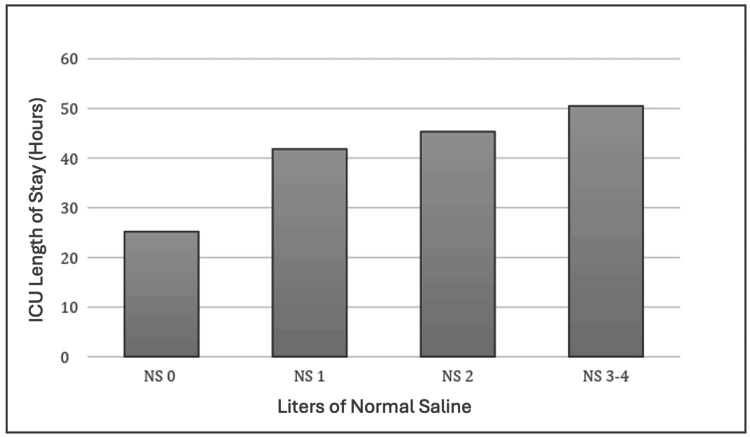
ICU length of stay and liters of normal saline administered One-way ANOVA (F = 1.113, p = 0.347). T-test 0 L of NS and 1 L of NS t(15,24) = -0.880, p = 0.192, 0 L of NS and 2 L of NS t(15,42) = -2.004, p = 0.0249, 0 L of NS and 3 or greater L of NS t(15,24) = -3.467, p = 0.00065. p-value considered significant at p<0.05. ANOVA: analysis of variance

There was no significant difference in ICU LOS between patients who received 0 L of NS, 1 L of NS, 2 L of NS, or 3 or greater L of NS using one-way ANOVA testing (F = 1.113, p = 0.347). There was no significant difference in ICU LOS between patients who received 0 L of NS and 1 L of NS t(15,24) = -0.880, p = 0.192. There was a significant difference in ICU LOS between patients who received 0 L of NS and 2 L of NS t(15,42) = -2.004, p = 0.0249 and patients who received 0 L of NS and 3 or greater L of NS t(15,24) = -3.467, p = 0.00065. Figure 1 demonstrates the results using ICU length of stay as the primary endpoint.

Secondary endpoint

Overall Length of Stay

**Figure 2:**
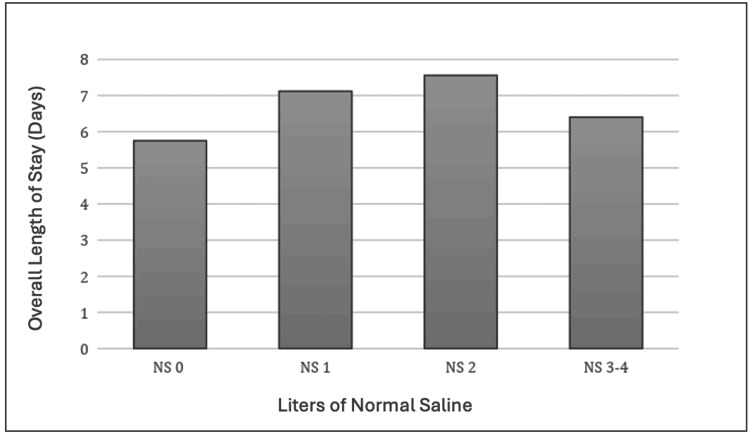
ICU length of stay and liters of normal saline administered One-way ANOVA testing (F = 0.203, p = 0.894). T-test 0L of NS and 1L of NS t(15,24) = -0.5278, p = 0.300, 0 L of NS and 2 L of NS t(15,42) = -0.6485, p = 0.260 and 0 L of NS and 3 or greater L of NS t(15,24) = -0.4689, p = 0.321. p-value considered significant at p<0.05. ANOVA: analysis of variance

There was no significant difference in overall LOS between patients who received 0 L of NS, 1 L of NS, 2 L of NS, or 3 or greater L of NS using one-way ANOVA testing (F = 0.203, p = 0.894). There was no significant difference in overall LOS between patients who received 0L of NS and 1L of NS t(15,24) = -0.5278, p = 0.300, 0 L of NS and 2 L of NS t(15,42) = -0.6485, p = 0.260 and 0 L of NS and 3 or greater L of NS t(15,24) = -0.4689, p = 0.321. Figure 2 demonstrates the results using this secondary endpoint.

Duration of Insulin Infusion After DKA Anion Gap Closure

**Figure 3:**
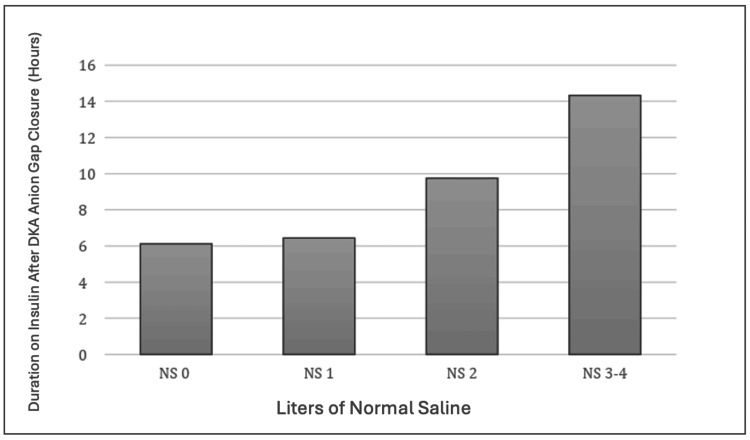
Liters of normal saline administered and duration on insulin infusion after DKA anion gap closure One-way ANOVA testing (F = 4.624, p = 0.004). T-test 0 L of NS and 1 L of NS t(15,24) = -0.1699, p = 0.433, 0 L of NS and 2 L of NS t(15,42) = -1.653, p = 0.0519, 0 L of NS and 3 or greater L of NS t(15,24) = -2.422, p = 0.0101. p-value considered significant at p<0.05. ANOVA: analysis of variance

There was a significant difference in the duration of insulin infusion after DKA anion gap closure between patients who received 0 L of NS, 1 L of NS, 2 L of NS, or 3 or greater L of NS using one-way ANOVA testing (F = 4.624, p = 0.004). There was no significant difference in the duration of insulin infusion after DKA anion gap closure between patients who received 0 L of NS and 1 L of NS t(15,24) = -0.1699, p = 0.433. There was no significant difference in the duration of insulin infusion after DKA anion gap closure between patients who received 0 L of NS and 2 L of NS t(15,42) = -1.653, p = 0.0519. There was a significant difference in the duration of insulin infusion after DKA anion gap closure between patients who received 0 L of NS and 3 or greater L of NS t(15,24) = -2.422, p = 0.0101. Figure 3 reports the duration of insulin infusion after DKA anion gap closure with regard to liters of NS administered.

Non-anion Gap Acidosis After DKA Anion Gap Closure

**Figure 4:**
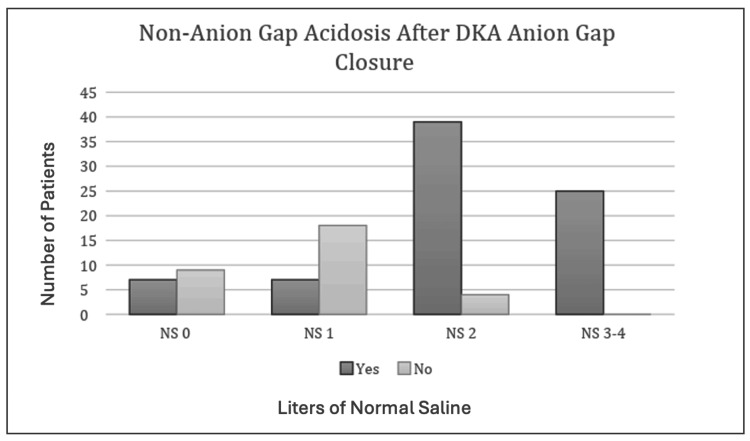
Patients with non-anion gap acidosis after DKA anion gap closure and liters of normal saline administered Chi-square test using chi-square (X2(3) = 47.063, p = 0.0000). p-value considered significant at p<0.05.

There was a significant difference in the number of patients who had a non-anion gap acidosis after DKA anion gap closure between patients who received 0 L of NS, 1 L of NS, 2 L of NS or 3 or greater L of NS using chi-square (X2(3) = 47.063, p = 0.0000). Figure 4 presents the number of patients with non-anion gap acidosis after DKA anion gap closure and the liters of NS administered.

## Discussion

This study showed that patients who received increased amounts of isotonic normal saline had longer ICU length of stay, increased duration of insulin infusion, and increased rates of non-anion gap metabolic acidosis after DKA anion gap closure. Numerous guidelines recommend initial resuscitation with isotonic normal saline (0.9% sodium chloride) [4]. Large-volume resuscitation with isotonic normal saline has been called into question due to studies that have demonstrated a statistically significant reduction in the incidence of iatrogenic hyperchloremia with the use of LR as compared to NS as fluid resuscitation in DKA [6]. Although the incidence of iatrogenic hyperchloremia may seem clinically insignificant, it is unclear if this led to an increased duration of time on insulin infusions and increased ICU and hospital length of stay, as demonstrated by our retrospective study.

One cluster randomized clinical trial comparing isotonic normal saline with balanced crystalloids (lactated Ringers) demonstrated a more rapid resolution of DKA [7]. This suggests that balanced crystalloids may be preferred over saline for the acute management of adults with DKA. We demonstrated a greater incidence of non-anion gap acidosis after DKA anion gap closure between patients who received 0 L of NS, 1 L of NS, 2 L of NS, or 3 or greater L of NS. As an extension of this, we also found an increase in the time on the insulin drip in patients who received large quantities of NS (greater than 3L). Finally, we found that patients who received 2 or more liters of isotonic normal saline had longer ICU length of stay when compared to patients who received 0 liters of isotonic normal saline, likely related to the ongoing non-anion gap metabolic acidosis. This suggests that larger volumes of isotonic saline used for initial resuscitation led to increased incidence of secondary hyperchloremia, non-anion gap metabolic acidosis, increased duration of insulin infusion, and increased ICU length of stay. Although we did not show an increased overall length of stay, it is worth noting that increased ICU length of stay leads to increased healthcare and patient costs. A typical ICU hospital stay costs 12,931 +/- 20,569 dollars for those not requiring mechanical ventilation [9,10]. Patients who received 2 liters of isotonic normal saline and those who received 3 or greater liters of isotonic normal saline had an average of 20.14 and 25.29 hours longer ICU stay, respectively, as compared to those who received 0 liters of isotonic normal saline for initial volume resuscitation. As DKA is a commonly seen disease pathology that requires ICU-level care, the impact on healthcare costs should not be underestimated [11,12]. The current study provides additional evidence that balanced crystalloids, such as lactated Ringers, should be used in lieu of normal saline for initial large-volume resuscitation in patients being treated for DKA.

## Conclusions

Numerous guidelines recommend isotonic normal saline as the initial fluid choice for large-volume resuscitation in patients with DKA. We demonstrated a greater incidence of non-anion gap acidosis after DKA anion gap closure, an increase in time on insulin drip in patients who received large quantities of NS (greater than 3L), and that patients who received 2 or more liters of isotonic NS had longer ICU length of stay when compared to patients who received 0 liters of isotonic NS. These results suggest that balanced crystalloids, such as lactated Ringers, should be used in lieu of normal saline for initial large-volume resuscitation in patients being treated for DKA.
